# The pattern of neonatal gastro-intestinal perforation in upper Egypt

**DOI:** 10.1186/s43159-020-00029-9

**Published:** 2020-06-25

**Authors:** Nezar Abd Elrouf Abo-Halawa, Mohamed Ahmed Negm, Mohamed Fathy

**Affiliations:** 1 Pediatric Surgery unit, Qena Faculty of Medicine, South Valley University, Qena, Egypt; 2 Pediatric Surgery Unit, Faculty of Medicine, Minia University, Minia, Egypt

**Keywords:** Neonatal gastro-intestinal perforation, Necrotizing enterocolitis, Perforation, Upper Egypt

## Abstract

**Background:**

Neonatal gastro-intestinal perforation [NGIP] is one of the major problems in pediatric surgical practice. Although the outcomes of neonatal surgery have improved markedly over the past decade the mortality rates of neonates with NGIP are still high. The aim of this study was to present the possible etiological factors, clinical findings, and operative procedures of NIP in our locality.

**Results:**

A total of 34 neonates with NGIP were included in this study. The median age at presentation was (15.8 ± 7.0 SD) days. The median interval between presentation and surgical interference was (2.0 ± 1.1 SD).Necrotizing enterocolitis [NEC] was the commonest cause of neonatal gastro-intestinal perforation. The commonest site of perforation was the colon [11cases]. The overall mortality rate was 11 cases [32.4%]. The main cause of mortality was neonatal NEC [6 cases]. Eight cases [40 %] died out of 20 cases which the interval between the presentation and interference were more than one day.

**Conclusions:**

Neonatal gastro-intestinal perforations are still associated with high mortality rate in our institutions, and delayed diagnosis with increased interval between the presentation and surgical intervention are associated with increased mortality. In our locality, although NEC is the commonest cause of NGIP, the iatrogenic cause is relatively higher than reported.

## Background

Neonatal gastro-intestinal perforation [NGIP] is one of the major problems facing pediatric surgeons worldwide [1]. Necrotizing enterocolitis [NEC] is still the major cause of NIP [2]. Although the outcomes of neonatal surgery have been improved markedly over the past decade due to the development of neonatal intensive management and care, as ventilator care, better surgical, and anesthetic techniques such as ventilator management, operative, and anesthetic techniques, the mortality rates of neonates with NIP are still high, ranging from 15 to 70%. This mortality depends on some causes such as birth weight, number of perforation, and delayed presentation [3–10]. However, early diagnosis and rapid transfer of these cases may have a good prognostic value.

The aim of this study was to present our experience of NGIP as the possible etiological factors, clinical findings and operative procedures in our locality at Upper Egypt. We also sought to investigate the relationship between the demographic characteristics data [gestational age, birth weight, the age of presentation and sex] as well as operative findings (causes of NGIP, sites, numbers of perforation and operative procedures done) with prognosis and survival rate of NIP. The identification of this relationship may enable early intervention, possibly leading to improved outcomes.

## Methods

After institutional review board approval, a retrospective review of all neonates with NGIP at Upper Egypt from October 2014 to April 2017 was performed. Data were collected from patient's charts including age, sex, gestational age, birth weight, age at presentation, clinical features on examination, interval between presentation and intervention, radiological finding (plain X-rays, contrast studies and ultrasound study), laboratory findings, causes and sites of perforations, types of operative procedures, and mortality and morbidity.

## Results

A total of 43 patients were managed during the period under review but 9 cases were excluded due to incomplete data. Thirty-four (34) neonates with neonatal gastro-intestinal perforation were included in this study. There were 19 male (55.9%) and 15 female (44.1%).

### Age

The average age at presentation was 15.8 ± 7.0 days, with a range of 3–28 days. Their birth weight ranged from 1500 to 3600 g. Gestational age of the subjects ranged from 30 to 40 weeks with an average of 36.03 ± 2.736. The presentation—surgical intervention interval was 1–6 days (SD 2.0±1.1) (Table 1).

**Table 1:** Patients’ demographic and clinical data

Demographics	Range	Mean ± SD
Gender		
• Male	20 (58.8%)	
• Female	14 (41.2%)	
Gestational age (weeks)	30-40	36.03±2.736
Age at presentation (days)	3-28	15.79±7.023
Weight (grams)	1500-3400	2541.18±492.438
The interval between presentation and interference (days)	1-6	1.97±1.141
The interval between presentation and interference (day)		
• 1day	13 (38.2%)	
• 2days	14 (41.3%)	
• 3 days	5 (14.7%)	
• 5 days	1 (2.9%)	
• 6 days	1 (2.9%)	

### Diagnosis of NGIP

Fifteen cases (44.1%) were diagnosed clinically and radiologically (plain abdominal X-rays and/or contrast study), 13 cases (38.2 %) were diagnosed by radiological studies, 5 cases (14.7%) were diagnosed by clinical examination with inconclusive radiological studies while one case was discovered during laparotomy done for exomphalos minor.

#### Causation

**Fig. 1:**
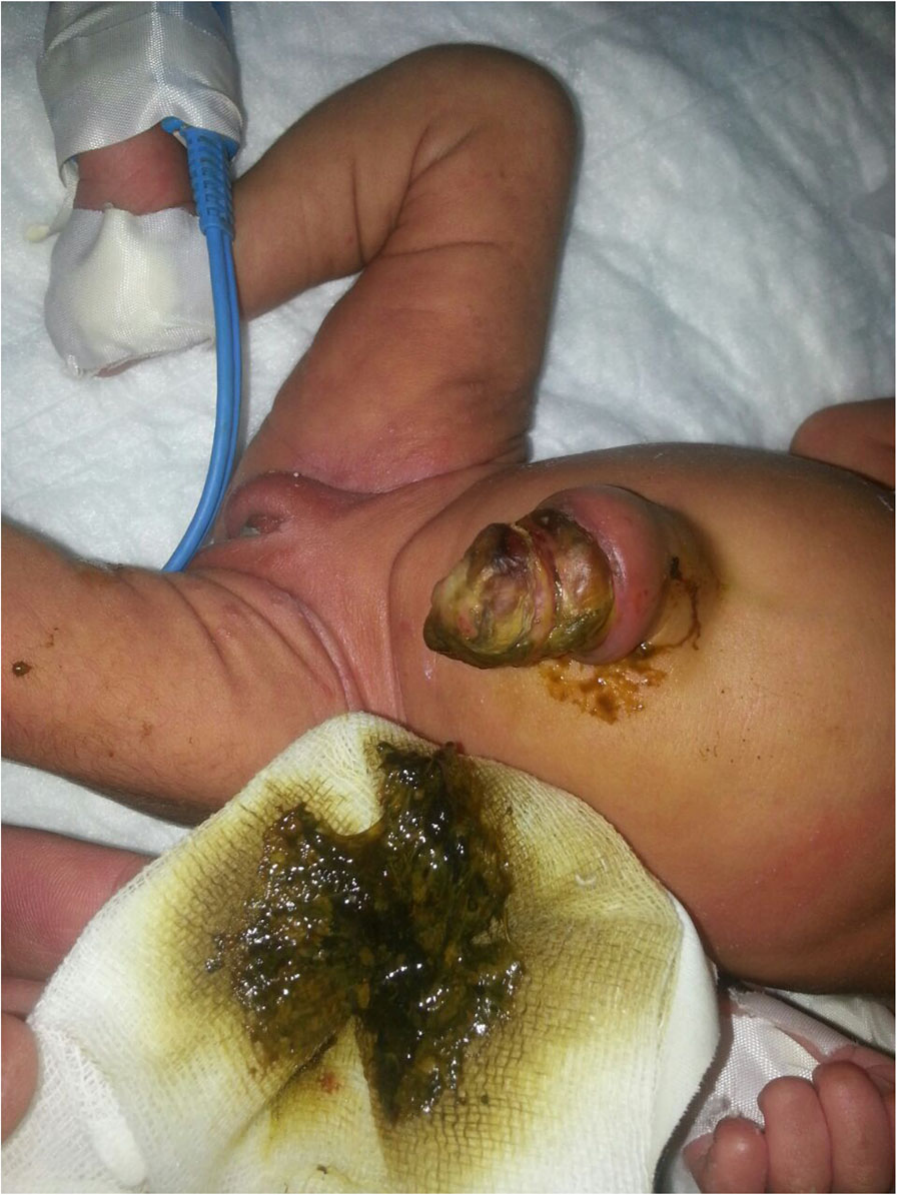
Exomphalos with perforated Meckel’s diverticulum

**Fig. 2:**
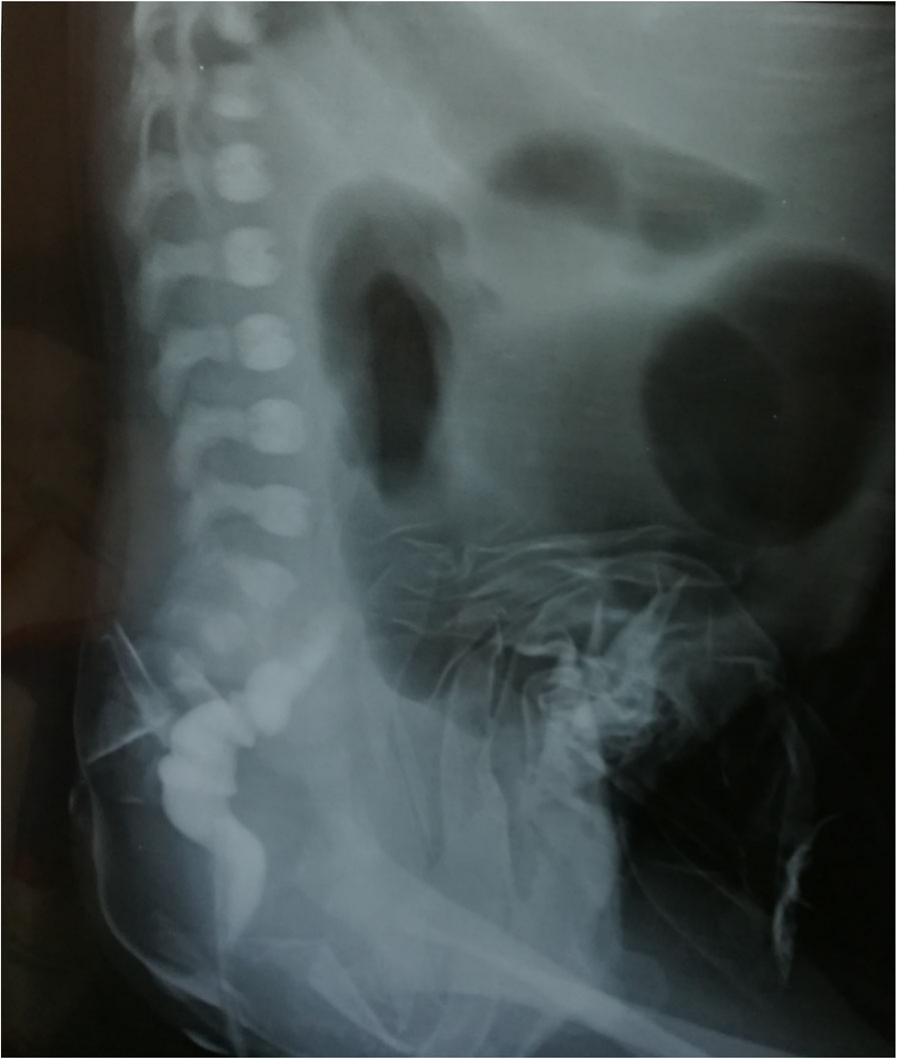
Gastrographin enema show rectal perforation with extravasation of contrast in the peritoneal cavity

In this study, necrotizing enterocolitis was the commonest cause of neonatal gastro-intestinal perforation with16 cases, followed by 6 iatrogenic perforations. Hirschsprung’s disease (HSD) was the cause of NGIP in 5 cases; 3 of them were iatrogenic while 2 were spontaneous. Neglected jejunoileal atresia resulted in NIP in 2 cases while one case was classified idiopathic. One case each resulted from complicated meconium peritonitis, Meckel’s diverticulum, neglected anorectal malformation and Exomphalos minor with perforated Meckel’s diverticulum (Fig. 1, Table 2) The causes of the 6 iatrogenic perforations were; rectal perforation after contrast enema in 2 cases (Fig. 2), rectal perforation after colonic washout in another 2 cases and gastric perforation after nasogastric tube insertion in 2 cases.

**Table 2:** Cause-related prognosis of gastro-intestinal perforations

Cause	Survival		Mortality		Total	
	*N*	%	*N*	%	*N*	%
NEC	10	29.4	6	17.6	16	47.1
Iatrogenic perforation	5	17.6	1	2.9	7	20.6
HSD	5	11.8	0	0	4	11.8
Exomphalos with perforated Meckel’s diverticulum	1	2.9	0	0	1	2.9
High ARM without fistula	0	0	1	2.9	1	2.9
Ileal atresia	0	0	1	2.9	1	2.9
Jejunal atresia	0	0	1	2.9	1	2.9
Meconium ileus with perforation	0	0	1	2.9	1	2.9
Idiopathic gastric perforation	1	2.9	0	0	1	2.9
Volvulus	1	2.9	0	0	1	2.9
Total	23	67.6	11	32.4	34	100

**Fig. 3:**
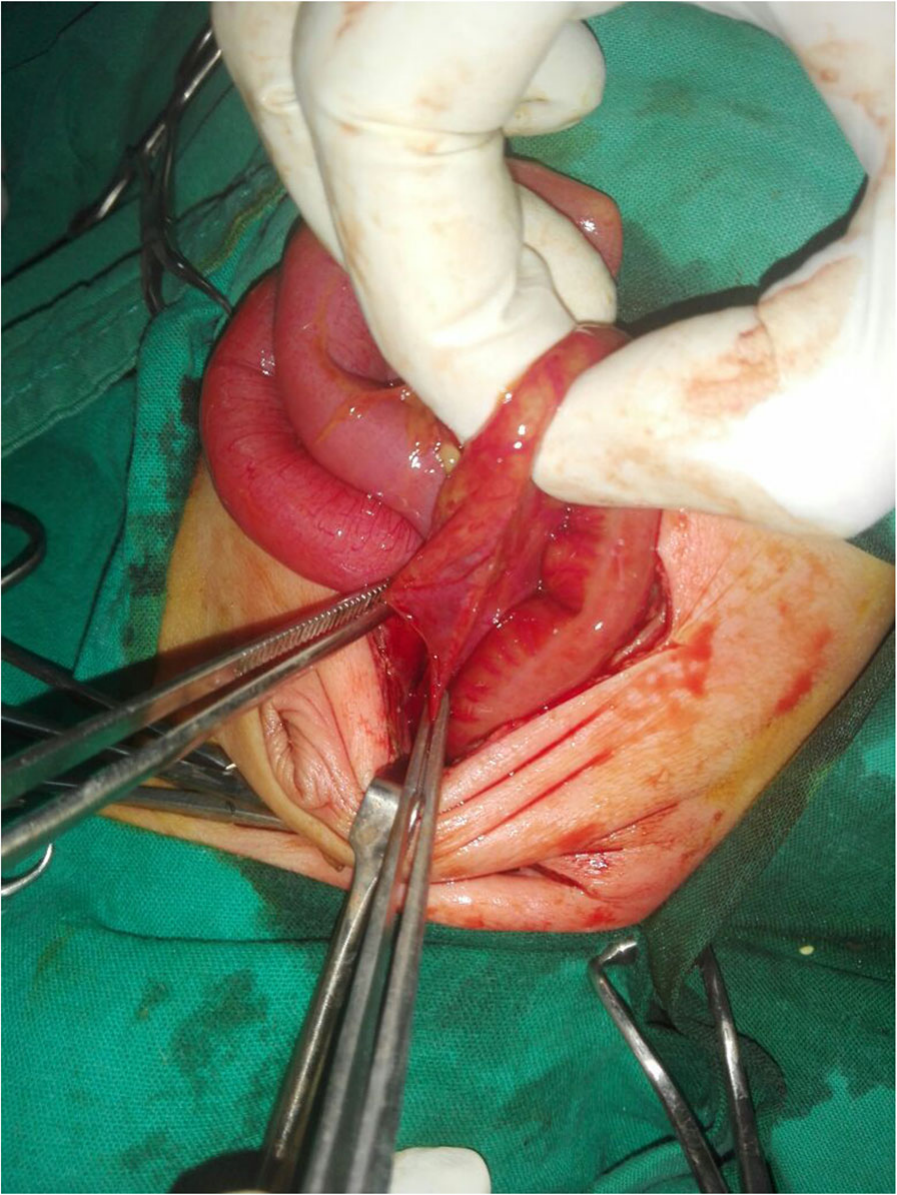
Anterior wall rectal perforation

**Fig. 4:**
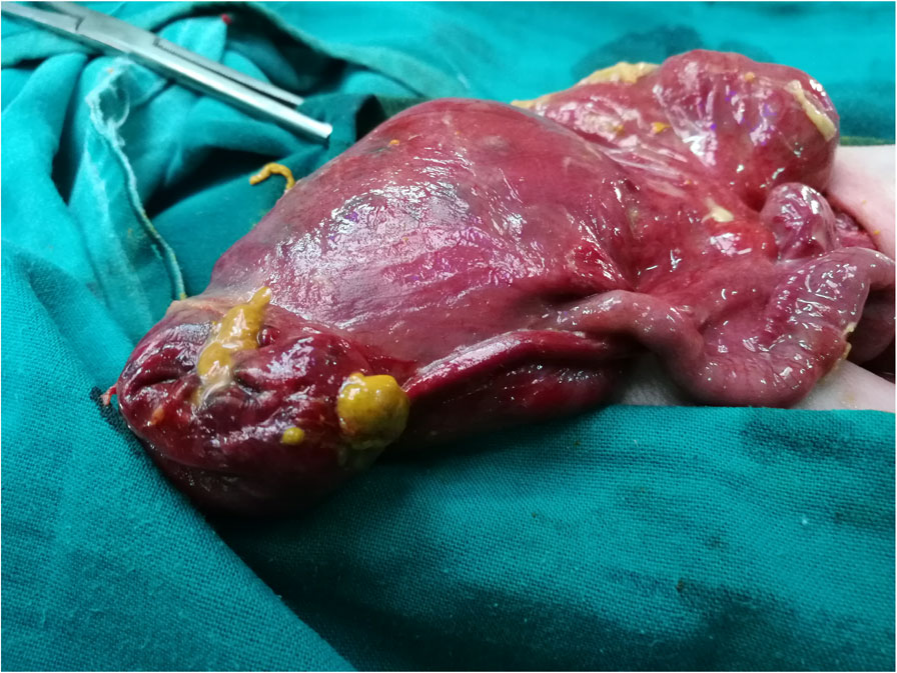
Jejunal perforation with jejunal atresia

The site of perforation was found in the colon in 11 cases followed by ileum in 8 cases, rectum in 5 cases (Fig. 3), stomach in 3 cases, jejunum in only one case (Fig. 4). Multiple perforations involving the ileum and colon were seen in 3 cases, the stomach and colon in 2 cases while it involved stomach and ileum in one case (Table 3).

**Table 3:** Sites related prognosis of gastro-intestinal perforations

Causes	Survival		Mortality		Total	
	*N*	%	*N*	%	*N*	%
Gastric	2	5.9	1	2.9	3	8.8
Jejunum	0	0	1	2.9	1	2.9
Stomach and colon	1	2.9	1	2.9	2	5.9
Stomach and ileum	0	0	1	2.9	1	2.9
Ileum	6	17.6	1	2.9	7	20.6
Ileum and colon	1	2.9	2	5.9	3	8.8
Meckel	1	2.9	0	0.0	1	2.9
Colonic	9	26.5	2	5.9	11	32.4
Rectum	3	8.8	2	5.9	5	14.7
Total	23	67.6	11	32.4	34	100

#### Treatment

The main line of treatment in this study was creation of a stoma. Colostomy was performed in 12 cases and ileostomy in 11 cases. Primary closure was performed in 6 patients; 3 in the stomach, 1 in the ileum, and 2 in the small bowel. Small bowel resection and anastomosis was done in only three cases while two cases were treated with primary peritoneal drainage. One of these patients died while the later underwent successful primary closure (Table 4).

**Table 4:** Surgical intervention and outcomes

Operative procedure	Survival		Mortality		Total	
	*N*	%	*N*	%	*N*	%
Colostomy	8	23.5	4	11.9	12	35.3
Ileostomy	10	29.5	1	2.9	11	32.4
Primary closure	3	8.8	3	8.8	6	17.6
Resection and anastomosis	1	2.9	2	5.9	3	8.8
primary peritoneal drainage	1	2.9	1	2.9	2	5.9
Total	23	67.6	11	32.4	34	100

#### Post-operative complications

Surgical site infection was recorded in 19 cases (55.9%), followed by sepsis in 16 (47.1%). Malnutrition occurred in 9 cases (26.5%) patients, skin excoriation in 6 cases (17.6%) and anastomotic leakage in 2 cases (5.9%). All dead 11 patients had postoperative sepsis. Five of these cases had sepsis with malnutrition, 4 had sepsis alone, 1 case had sepsis, malnutrition, and leakage with burst abdomen while in 1 case, and there was sepsis with anastomotic leakage.

#### Mortality

The overall mortality rate was 11 cases (32.4%). The main causes of mortality were NEC [6 cases], jejunoileal atresia [2 cases], ARM [1 case], rectal injury [1 case], and meconium peritonitis [1 case].

#### Gestational age (Table 5)

Out of 10 cases with low birth weight (1500–2500 gm), 7 cases (70%) died. The only case of a very low birth weight (1300 gm) also died. However, only 3 babies (13%) of 23 babies with birth weight > 2500 gm died. Prematurity was therefore strongly correlated with death (*P* value = 0.002).

**Table 5:** The relationship between the demographic characteristics and prognosis

Demographics	Survived *N*(%) 23 (100%)	Died *N*(%) 11 (100%)	Total *N*(%) 34 (100%)	*P*-value
Gender				
Male	14 (60.9%)	6 (54.5%)	20 (58.8%)	0.7
Female	9 (39.1%)	5 (45.5%)	14 (41.2%)	
Gestational age				
Preterm	9 (39.1%)	8 (72.7%)	17 (50%)	0.002
Full term	14 (60.9%)	3 (27.3%)	17 (50%)	
Age at presentation				
10 days or less	4 (17.4%)	4 (36.4%)	8 (23.5%)	0.06
More than 10 days	19 (82.6%)	7 (63.6%)	26 (76.5%)	
Weight				
Low birth weight	3 (13 % )	7 (63.6%)	10 (41.2%)	≤0.001
Normal birth Wight	20 (87 %)	4 (36.4%)	24 (58.8%)	
The interval between presentation and intervention				
Less than 1 day	11 (47.8%)	3 (27.3%)	14 (41.2%)	0.016
More than 1 day	12 (52.2%)	8 (72.7%)	20 (58.8%)	

#### Age of presentation

Of 9 cases who presented at age 10 days or less, 4 cases (44.4%) died with -ve correlation (*P* value 0.06) while out of 25 cases with age at presentation more than 10 days, 7 cases (28%) cases died (Table 5), the difference was not statistically significant.

#### Presentation-intervention interval

Twenty cases had surgical intervention more than 1 day. Eight cases (40%) of these patients died. Only 3 cases (27.3%) of 11 babies who presented within 24 h died. Intervention time of more than 1 day was significantly associated with high mortality rate (*P* value = 0.016).

#### No of perforations

Multiple perforations were strongly associated with high mortality rate in this study (*P* value = 0.01) (Table 6).

**Table 6:** Study outcome according to the number of perforations

Number of perforation	Survived		Died		Total		*P* value
	*N*	%	*N*	%	*N*	%	
1	22	64.7	6	17.6	28	82.4	0.01
2	1	2.9	3	8.8	4	11.8	
Multiple	0	0	2	5.9	2	5.9	
Total	23	67.6	11	32.4	34	100	

## Discussion

Early identification of infants with NGIP may lead to prompt treatment with sure benefit to the outcome. All surgical neonatal emergencies depend mainly on the efficacy of the bases of surgical care which was provided by hospitals and surgeons. It has been known for some time that the level and volume of neonatal intensive care at the hospital of birth strongly influences mortality rate of neonates in need of surgical intervention [11–14].

Proper referral and transportation also had its impact on the chances of neonate’s prognosis following emergency surgery [15]. This fact is very important in our locality as only two centers have adequate facilities for neonatal surgical intervention for NGIP. The results of this study showed that delayed diagnosis and transfer are of the most important causes of increased mortality. In the same way, the increased interval between the presentation and intervention may lead to high mortality rate.

This may be attributed to lack of clinical experience for early diagnosis, lack of communication between pediatricians and pediatric surgeons, in addition to delay in transfer of babies after diagnosis to our institutions. This mandates continuous communication between pediatricians and pediatric surgeons to improve clinical experience, early diagnosis, and management. Moreover, good training is required for paramedical and nursing staff dealing with neonates.

In cases with NEC, pneumoperitoneum is the most relevant radiological indicator of bowel perforation that may need surgical intervention. However, pneumoperitoneum is present in less than half of all infants with gastro-intestinal perforation or necrosis at the time of operative exploration [16]. In our study, five cases were diagnosed by clinical examination with inconclusive radiological studies, also two cases of died from NEC, the radiological X-ray is insignificant, and transport of babies was delayed, so it is important to increase the clinical sense and raise suspicion and other diagnostic modalities for diagnosis of perforation in NEC.

It is reported that necrotizing enterocolitis is the most devastating and frequent surgical emergency in the neonatal intensive care units (NICUs), occurring in 0.7 per 1000 patients, and in up to 7% of those hospitalized in NICU. An estimated 20 to 40% of infants with NEC will need surgical interference, and the mortality rate in these infants can be as high as 50% [17–20]. In our study, NEC is the commonest cause of death [54.5%] with high incidence in preterm cases, which is similar to other studies [9, 21]. In the current study, necrotizing enterocolitis is the commonest cause of NGIP (47.1%), which is similar to those reported by others [1, 4, 6, 21–23].

According to the result of this study, there are relatively high percentage of the iatrogenic perforation (sex cases), and most of the cases were caused by inexperienced staff during contrast enema or rectal wash, which indicate the need of adequate training and good supervision to decrease these causes, this is similar to result detect by Elhalaby et al. who reported also a relatively high frequency of iatrogenic colorectal perforations [1].

In the present study, the prognosis was better in large bowel perforation more than small bowel perforation, and this is similar to other studies [21–23]. This result can correlate with line of treatment as the prognosis is better in neonate underwent colostomy rather than other line of treatment especially ileostomy and resection with anastomosis. This finding can be explained by the fact that colostomy reduces the time of surgery, and early postoperative feeding which is important in our institutes due to lack of availability of total parental nutrition.

Mortality from NGIP is still high, although advancements in anesthesia and neonatal intensive care, the high mortality has to increase extremely premature babies [7, 24]. In this study, mortality rate is high in prematurity, low birth weight, multiple perforations, and delayed presentation which was similar to what reported by other authors [1, 4, 6, 8, 12, 21, 23].

## Conclusion

Neonatal gastro-intestinal perforations are still associated with high mortality rate in our institutions, delayed diagnosis with increased interval between the presentation and surgical intervention are associated with increased mortality. In our locality, although NEC is the commonest cause of NGIP, the iatrogenic cause is relatively higher than reported.

## Data Availability

The datasets used and/or analyzed during the current study are available from the corresponding author on reasonable request.

## Abbreviations

NGIP: Neonatal gastro-intestinal perforation
NEC: Necrotizing enterocolitis
HSD: Hirschsprung’s disease
ARM: Anorectal malformations
